# Acute toxicity dataset for QSAR modeling and predicting missing data of six pesticides

**DOI:** 10.1016/j.dib.2020.105150

**Published:** 2020-01-17

**Authors:** Ya-Qian Xu, Shu-Shen Liu, Bing-Qing Lu, Ze-Jun Wang

**Affiliations:** a State Key Laboratory of Pollution Control and Resource Reuse, College of Environmental Science and Engineering, Tongji University, Shanghai, 200092, PR China; b Yangtze River Water Environment, Ministry of Education, College of Environmental Science and Engineering, Tongji University, Shanghai, 200092, PR China; c Shanghai Institute of Pollution Control and Ecological Security, Shanghai, 200092, PR China

**Keywords:** Water quality criteria, Non-linear curve fitting, Toxicity prediction, Variable selection and modeling, Variable importance in projection

## Abstract

This data article presents 1) the acute toxicity (LC_50_ or EC_50_ (μg⋅L^−1^)) values of various chemicals for ten species, which were used to develop ten robust quantitative structure–activity relationship (QSAR) models, 2) the values of the various descriptors in the ten QSAR models, and 3) the acute toxicity values of six pesticides (acetochlor, chlorpyrifos, dimethoate, glyphosate, malathion, and paraquat) for various species, which were applied to establish species sensitivity distribution (SSD) models. The provided LC_50_ or EC_50_ (μg⋅L^−1^) data were collected from the PAN pesticide database and the United States Environmental Protection Agency ecotoxicology database and/or were predicted by the QSAR models. The values of the descriptors in the ten QSAR models were based on the optimal descriptors computed by the DRAGON software (version 7) and subsequently optimized by partial least squares modeling. All the data included in this manuscript are related to the research titled, “Conlecs: A novel procedure for deriving the concentration limits of chemicals outside the criteria of human drinking water using existing criteria and species sensitivity distribution based on quantitative structure–activity relationship prediction” [1].

**Table:** Specification table

Subject area	Environmental science
Specific subject	Ecotoxicology
Type of data	Tables
How data were acquired	The PAN pesticide database and the United States Environmental Protection Agency ecotoxicology database
Data format	Raw and predicted
Parameters for data collection	The data depend on the PAN and EPA databases
Description of data collection	The collected data represents the acute toxicities of different chemicals for ten species and the toxicities of six pesticides for various species
Data source location	http://www.pesticideinfo.org/; http://www.epa.gov/ecotox/
Data accessibility	Data are with this article
Related research article	Lu, B.Q., Liu, S·S., Wang, Z.J., Xu, Y.Q. Conlecs: A novel procedure for deriving the concentration limits of chemicals outside the criteria of human drinking water using existing criteria and species sensitivity distribution based on quantitative structure-activity relationship prediction. Journal of Hazard Materials, 2019, 384: 121,380 [1]

**Table:** 

**Value of the data** • These data can be used to develop a robust quantitative structure–activity relationship (QSAR) model, which can predict the toxicity of a chemical to certain species without any experimental toxicity data and facilitate the establishment of a uniform species sensitivity distribution (SSD) model to derive water quality criteria. • Researchers and water policy makers can benefit from these data for assessing the ecological risk of chemicals. Besides saving the time, cost, and effort of determining the toxicities of some chemicals, these data can also be used to expand and optimize an SSD model to increase its precision and universality. • The developed QSAR model can also be used to assess the reliability of contradictory experimental data. In addition, QSAR predicted data can complement the toxicity data of a given chemical for a specific species, and thereby increase the robustness of the corresponding SSD model. • The QSAR models based on ten species can also predict the toxicity of a different chemical, which is not within the scope of this study. In addition, the descriptors in the models can explain why a molecular structure increases/reduces chemical toxicity.

## 1 Data

Ten QSAR models of toxicities of various chemicals to ten species were developed by the partial least squares based on the variable importance in projection (VIPLS) and variable selection and modeling based on prediction (VSMP) procedures. Further, six SSD models of the toxicities of six chemicals to various species were constructed from their toxicity data for different species (Table 1a, Table 1b, Table 1c, Table 1d, Table 1e, Table 1f, Table 1g, Table 1h, Table 1i, Table 1j, Table 2a, Table 2b, Table 2c, Table 2d, Table 2e, Table 2f).

**Table 1a:** Values of the toxicities (LC_50_/EC_50_) and descriptors (*X*_*i,j*_)* of various chemicals to ten species, where *X*_*i,j*_ represents the *j*th descriptor in the *i*th QSAR model (*i* = 1,2,3, …,10; *j* = 1,2, …,8). Toxicities of 35 chemicals to *X. laevis.*

No.	Chemical	LC_50_/EC_50_ (μg⋅L^−1^)	*X*_*1,1*_	*X*_*1,2*_	*X*_*1,3*_	*X*_*1,4*_	*X*_*1,5*_	*X*_*1,6*_	Resource**
1	Chlorpyrifos (CHL)	2.80E+03	1.77E+02	1.80E+02	3.36E+02	3.37E+02	3.86E+02	3.89E+02	ECOTOX
2	Glyphosate (GLY)	9.02E+03	0.00E+00	−4.45E-01	−9.74E-01	−4.69E-01	2.30E+00	1.89E+00	ECOTOX
3	Malathion (MAL)	1.09E+04	3.43E+00	−1.88E+00	4.18E-01	8.11E-01	1.55E+00	1.66E+00	ECOTOX
4	Paraquat (PAR)	2.33E+03	0.00E+00	−2.81E-01	−5.45E-01	−1.57E+00	4.82E+00	1.26E+00	ECOTOX
5	Acetochlor (ACE)	2.71E+03	4.41E+00	−1.00E+00	−2.23E+00	−6.15E-01	1.62E+00	1.21E+00	ECOTOX
6	Aniline	2.89E+05	1.00E+00	0.00E+00	−1.96E+00	−3.61E-01	1.77E+00	6.56E-01	ECOTOX
7	Atrazine	9.43E+04	1.00E+00	0.00E+00	−3.89E-01	−1.10E-01	2.50E-01	9.07E-01	ECOTOX
8	Dieldrin	7.10E+01	1.00E+00	0.00E+00	−3.89E-01	−1.10E-01	2.50E-01	9.07E-01	ECOTOX
9	Flumorph	8.32E+04	1.80E+01	4.24E-01	−5.50E-02	−1.29E-01	4.01E+00	7.92E+00	ECOTOX
10	Pyraclonil	2.63E+03	1.00E+00	1.00E+00	−3.80E+00	−2.42E+00	3.91E+00	9.38E-01	ECOTOX
11	Permethrin	4.58E+02	1.00E+00	7.62E-01	−1.58E+00	−1.43E+00	6.20E+00	1.62E+00	ECOTOX
12	Pentachlorophenol	2.07E+02	1.00E+00	1.09E+00	−2.00E+00	2.25E-01	5.10E+00	1.04E+00	ECOTOX
13	Diuron	2.91E+04	8.41E+00	−1.30E+00	7.58E-01	8.72E-01	7.36E-01	7.78E+00	ECOTOX
14	Benzo [*a*]pyrene	1.31E+04	6.41E+00	−1.00E+00	−4.08E-01	−6.20E-01	2.62E+00	3.44E+00	ECOTOX
15	Fluorouracil	6.50E-01	1.00E+00	1.11E+00	−7.95E-01	3.86E-01	2.25E+00	4.92E-01	ECOTOX
16	2-acetamidofluorene	9.77E+04	2.00E+00	−2.00E+00	−1.18E+00	1.96E+00	4.16E+00	1.69E+01	ECOTOX
17	Nicotine	5.22E+04	3.16E+00	−2.35E-01	−1.11E-01	−1.23E-01	2.71E+00	1.05E+00	ECOTOX
18	Caffeine	2.50E-01	2.00E+00	−1.00E+00	2.86E-01	−3.36E-01	1.40E+00	1.34E+00	ECOTOX
19	Trichloroethylene	4.34E+04	8.41E+00	−1.19E+00	−2.51E+00	−9.30E-02	9.09E+00	6.24E+00	ECOTOX
20	Saccharin	1.53E+01	1.00E+00	0.00E+00	1.78E-01	3.69E-01	0.00E+00	0.00E+00	ECOTOX
21	Naphthalene	2.10E+03	8.16E+00	−1.00E+00	4.08E-01	4.06E-01	2.23E+00	1.97E+01	ECOTOX
22	Malaoxon	4.02E+02	6.16E+00	−1.62E+00	−4.38E-01	−3.97E-01	2.99E-01	7.12E-01	ECOTOX
23	Piperonyl butoxide	6.80E+04	3.00E+00	−2.81E-01	−1.11E+00	−7.25E-01	6.17E+00	1.38E+00	ECOTOX
24	Phenol	5.11E+04	0.00E+00	1.00E+00	−1.37E+00	−1.69E+00	4.04E+00	4.27E-01	ECOTOX
25	(e)-2-pentenoic acid	3.18E+06	1.00E+00	0.00E+00	−1.16E+00	4.60E-02	1.54E-01	1.28E+00	ECOTOX
26	(e)-3-hexenoic acid	1.50E+06	0.00E+00	0.00E+00	1.00E+00	−3.91E-01	9.95E-01	9.98E-01	PAN
27	2,4,6-trichlorophenol	1.20E+03	0.00E+00	0.00E+00	1.07E+00	−2.97E-01	1.19E+00	7.56E-01	PAN
28	2,2,2-trichloroethanol	7.80E+05	6.41E+00	−1.58E+00	6.72E-01	4.70E-01	7.29E-01	5.65E+00	PAN
29	Benzo(*a*)pyrene	1.31E+04	3.43E+00	0.00E+00	6.25E-01	−1.06E+00	0.00E+00	2.42E+00	PAN
30	Butyric acid	3.56E+06	1.00E+00	1.11E+00	−7.95E-01	3.86E-01	2.25E+00	4.92E-01	PAN
31	Hydroxyurea	1.22E+06	0.00E+00	0.00E+00	7.53E-01	−3.51E-01	1.42E+00	2.96E+00	PAN
32	(e)-2-octenoic acid	9.32E+04	3.41E+00	0.00E+00	1.65E+00	−1.05E+00	3.60E-01	1.88E+00	PAN
33	(e)-2-hexenoic acid	8.22E+05	0.00E+00	−1.90E+00	1.48E+00	−9.59E-01	2.57E+00	9.37E-01	PAN
34	Acridine	4.02E+03	0.00E+00	0.00E+00	1.18E+00	−5.14E-01	1.61E+00	1.06E+00	PAN
35	Dicrotophos	1.00E+05	7.16E+00	−4.45E-01	−1.30E+00	−9.87E-01	9.29E-01	5.46E-01	PAN

*X_1,1_ = Chi1_EA(ed), *X_1,2_* = SM03_AEA(dm), *X_1,3_* = Mor27s, *X_1,4_* = Mor28s, *X_1,5_* = H4s, and *X_1,6_* = HATS4s; ** ECOTOX and PAN denote that the toxicity of the corresponding chemical is from the ECOTOX and PAN databases, respectively.

**Table 1b:** Values of the toxicities (LC_50_/EC_50_) and descriptors (*X*_*i,j*_)* of various chemicals to ten species, where *X*_*i,j*_ represents the *j*th descriptor in the *i*th QSAR model (*i* = 1,2,3, …,10; *j* = 1,2, …,8). Toxicities of 40 chemicals to *B. b. japonicus.*

No.	Chemical	LC_50_/EC_50_ (μg⋅L^−1^)	*X*_*2,1*_	*X*_*2,2*_	*X*_*2,3*_	*X*_*2,4*_	*X*_*2,5*_	*X*_*2,6*_	*X*_*2,7*_	*X*_*2,8*_	Resource**
1	Paraquat (PAR)	7.22E+03	1.65E+02	0.00E+00	3.70E+00	−9.29E-01	0.00E+00	1.00E+01	6.00E+00	0.00E+00	ECOTOX
2	1,2-benzenedicarboxylic acid	3.79E+03	1.83E+02	0.00E+00	2.82E+00	−4.73E-01	2.00E+00	4.00E+00	6.00E+00	0.00E+00	ECOTOX
3	4-(4-chloro-2-methylphenoxy)butanoic acid ethyl ester	5.00E+03	2.86E+02	0.00E+00	2.71E+00	−1.16E+00	0.00E+00	7.00E+00	5.00E+00	6.00E+00	ECOTOX
4	Dazomet	4.00E+04	1.18E+02	0.00E+00	2.95E+00	−4.77E-01	0.00E+00	6.00E+00	0.00E+00	0.00E+00	ECOTOX
5	Dichlobenil	1.67E+04	1.14E+02	1.66E+01	2.94E+00	−2.34E-01	0.00E+00	3.00E+00	7.00E+00	0.00E+00	ECOTOX
6	Dialifor	1.20E+03	3.03E+02	0.00E+00	1.92E+00	−8.20E-02	2.00E+00	1.00E+01	6.00E+00	2.00E+00	ECOTOX
7	Carbendazim	4.00E+04	1.58E+02	1.33E+01	2.78E+00	−3.56E-01	0.00E+00	6.00E+00	0.00E+00	0.00E+00	ECOTOX
8	Cycloate	3.20E+03	3.88E+02	0.00E+00	5.85E+00	9.85E-01	0.00E+00	0.00E+00	1.20E+01	2.40E+01	ECOTOX
9	Chloromethane sulfonamide	4.00E+04	2.72E+02	0.00E+00	3.50E+00	−7.57E-01	0.00E+00	5.00E+00	5.00E+00	4.00E+00	ECOTOX
10	Chlomethoxyfen	4.00E+04	1.07E+02	0.00E+00	1.84E+00	−8.74E-01	0.00E+00	0.00E+00	0.00E+00	0.00E+00	ECOTOX
11	Chlorpropham	8.60E+03	2.57E+02	0.00E+00	2.51E+00	−4.50E-01	0.00E+00	9.00E+00	5.00E+00	7.00E+00	ECOTOX
12	Fluoronitrofen	1.40E+04	2.05E+02	1.80E+01	3.08E+00	−6.99E-01	0.00E+00	5.00E+00	6.00E+00	0.00E+00	ECOTOX
13	Difenphos	1.80E+03	2.11E+02	0.00E+00	3.53E+00	−1.05E+00	0.00E+00	6.00E+00	6.00E+00	6.00E+00	ECOTOX
14	Diquat dibromide	2.74E+05	1.99E+02	3.44E+01	3.72E+00	−1.73E+00	0.00E+00	2.00E+00	5.00E+00	2.00E+00	ECOTOX
15	Meptyldinocap	1.50E+02	4.20E+02	0.00E+00	4.53E+00	−1.42E+00	3.00E+00	4.00E+00	1.10E+01	1.10E+01	ECOTOX
16	Metolcarb	4.00E+04	1.54E+02	1.80E+01	2.69E+00	−7.16E-01	0.00E+00	7.00E+00	6.00E+00	0.00E+00	ECOTOX
17	Isoxathion	1.20E+04	2.19E+02	0.00E+00	3.51E+00	−2.03E+00	0.00E+00	1.00E+01	7.00E+00	1.00E+00	ECOTOX
18	MCPA	1.00E+04	2.12E+02	0.00E+00	2.47E+00	−8.66E-01	0.00E+00	5.00E+00	5.00E+00	0.00E+00	ECOTOX
19	MCPE	2.15E+04	2.11E+02	0.00E+00	2.96E+00	−8.21E-01	0.00E+00	7.00E+00	5.00E+00	0.00E+00	ECOTOX
20	MCPA-thioethyl	5.50E+03	2.28E+02	0.00E+00	2.44E+00	−4.70E-01	0.00E+00	7.00E+00	6.00E+00	2.00E+00	ECOTOX
21	Isothioate	7.30E+03	2.14E+02	0.00E+00	3.89E+00	−8.07E-01	0.00E+00	1.10E+01	3.00E+00	0.00E+00	ECOTOX
22	Isoprocarb	2.30E+04	2.01E+02	1.80E+01	3.27E+00	−8.87E-01	0.00E+00	7.00E+00	8.00E+00	2.00E+00	ECOTOX
23	HexachloroBenzene	4.20E+03	2.35E+02	0.00E+00	2.89E+00	−3.23E-01	0.00E+00	0.00E+00	0.00E+00	3.00E+00	ECOTOX
24	Captan	2.00E+02	2.62E+02	0.00E+00	3.59E+00	1.78E+00	0.00E+00	2.00E+00	9.00E+00	6.00E+00	ECOTOX
25	Folpet	6.50E+02	2.15E+02	0.00E+00	3.13E+00	1.23E+00	2.00E+00	4.00E+00	9.00E+00	6.00E+00	ECOTOX
26	Paraquat dichloride	7.19E+03	1.65E+02	0.00E+00	3.69E+00	−9.29E-01	0.00E+00	1.00E+01	6.00E+00	0.00E+00	ECOTOX
27	Promecarb	2.80E+04	2.24E+02	1.80E+01	2.92E+00	−9.96E-01	0.00E+00	6.00E+00	1.10E+01	4.00E+00	ECOTOX
28	Pebulate	6.10E+03	2.72E+02	0.00E+00	3.85E+00	−5.85E-01	0.00E+00	6.00E+00	2.00E+00	2.00E+00	ECOTOX
29	Propaphos	1.40E+04	2.88E+02	0.00E+00	3.70E+00	−2.75E+00	0.00E+00	1.10E+01	4.00E+00	0.00E+00	ECOTOX
30	Orbencarb	2.55E+03	2.53E+02	0.00E+00	3.67E+00	−5.66E-01	0.00E+00	1.00E+01	7.00E+00	1.00E+00	ECOTOX
31	Oxadiazon	2.80E+03	3.37E+02	0.00E+00	3.25E+00	−1.38E+00	0.00E+00	3.00E+00	6.00E+00	5.00E+00	ECOTOX
32	Molinate	2.18E+04	3.69E+02	0.00E+00	2.59E+00	−1.25E+00	0.00E+00	9.00E+00	1.00E+00	6.00E+00	PAN
33	Butoxycarboxim	4.00E+04	2.25E+02	0.00E+00	2.47E+00	−3.67E-01	0.00E+00	6.00E+00	3.00E+00	2.00E+00	PAN
34	Binapacryl	3.50E+02	2.34E+02	1.80E+01	2.67E+00	−4.24E-01	0.00E+00	7.00E+00	0.00E+00	0.00E+00	PAN
35	Butamifos	3.30E+03	3.50E+02	0.00E+00	3.73E+00	−1.17E+00	3.00E+00	3.00E+00	9.00E+00	6.00E+00	PAN
36	Oxycarboxin	4.00E+04	2.22E+02	1.80E+01	2.99E+00	−1.55E+00	3.00E+00	9.00E+00	4.00E+00	0.00E+00	PAN
37	Butachlor	1.80E+03	3.81E+02	0.00E+00	3.08E+00	−2.02E+00	0.00E+00	7.00E+00	9.00E+00	6.00E+00	PAN
38	Benfluralin	1.10E+04	2.90E+02	0.00E+00	3.96E+00	−1.99E+00	0.00E+00	6.00E+00	2.00E+00	5.00E+00	PAN
39	Asulam	1.10E+04	1.63E+02	1.64E+01	2.37E+00	−8.89E-01	0.00E+00	7.00E+00	2.00E+00	0.00E+00	PAN
40	Alachlor	3.17E+03	3.11E+02	0.00E+00	3.12E+00	−1.44E+00	0.00E+00	8.00E+00	8.00E+00	6.00E+00	PAN

*X_2,1_ = P_VSA_i_3, *X_2,__2_* = P_VSA_s_5, *X_2,3_* = VE1_RG, *X_2,4_* = Mor08 m, *X_2,5_* = nCconj, *X_2,6_* = H-047, *X_2,7_* = CATS2D_02_LL, and *X_2,8_* = CATS2D_05_LL; ** ECOTOX and PAN denote that the toxicity of the corresponding chemical is from the ECOTOX and PAN databases, respectively.

**Table 1c:** Values of the toxicities (LC_50_/EC_50_) and descriptors (*X*_*i,j*_)* of various chemicals to ten species, where *X*_*i,j*_ represents the *j*th descriptor in the *i*th QSAR model (*i* = 1,2,3, …,10; *j* = 1,2, …,8). Toxicities of 27 chemicals to *C. magister.*

No.	Chemical	LC_50_/EC_50_ (μg⋅L^−1^)	*X*_*3,1*_	*X*_*3,2*_	*X*_*3,3*_	*X*_*3,4*_	*X*_*3,5*_	Resource**
1	Malathion (MAL)	4.00E+01	2.00E+00	1.90E+01	0.00E+00	5.05E+00	0.00E+00	ECOTOX
2	1-methylnaphthalene	1.90E+03	1.00E+00	1.60E+01	0.00E+00	4.99E+00	0.00E+00	ECOTOX
3	Mesitylene	4.30E+03	1.00E+00	1.50E+01	0.00E+00	5.00E+00	0.00E+00	ECOTOX
4	1,2,4-trimethylbenzene	5.10E+03	1.00E+00	1.90E+01	0.00E+00	5.99E+00	0.00E+00	ECOTOX
5	1,2,4,5-tetramethylbenzene	2.10E+03	3.00E+00	4.30E+01	4.62E+01	1.30E+01	4.00E+00	ECOTOX
6	Metiram	5.90E+03	1.00E+00	9.00E+00	0.00E+00	1.99E+00	0.00E+00	ECOTOX
7	Trifluralin	3.07E+02	1.00E+00	1.00E+01	0.00E+00	2.99E+00	0.00E+00	ECOTOX
8	Benzene	1.08E+04	2.00E+00	2.90E+01	6.76E+01	6.29E+00	6.00E+00	ECOTOX
9	Toluene	2.80E+04	5.00E+00	4.10E+01	1.80E+01	1.41E+01	0.00E+00	ECOTOX
10	Dichlone	3.80E+01	2.00E+00	3.40E+01	2.51E+01	5.43E+00	5.00E+00	ECOTOX
11	Dieldrin	2.37E+01	2.00E+00	3.30E+01	4.19E+01	3.73E+00	1.00E+00	ECOTOX
12	Carbaryl	9.50E+00	1.00E+00	2.90E+01	6.87E+01	3.80E+00	0.00E+00	ECOTOX
13	Carbendazim	7.60E+03	2.00E+00	3.80E+01	2.51E+01	8.24E+00	4.00E+00	ECOTOX
14	2,4-D	3.16E+04	2.00E+00	2.00E+01	0.00E+00	5.06E+00	0.00E+00	ECOTOX
15	2-methylnaphthalene	1.30E+03	1.00E+00	2.90E+01	2.99E+01	4.30E+00	2.00E+00	ECOTOX
16	Propanil	6.43E+03	0.00E+00	4.60E+01	2.40E+01	1.18E+01	3.00E+00	ECOTOX
17	Selenium dioxide	7.38E+02	0.00E+00	2.00E+00	0.00E+00	1.39E+01	0.00E+00	ECOTOX
18	Methoxychlor	6.50E+00	2.00E+00	5.90E+01	0.00E+00	8.28E+00	2.00E+00	ECOTOX
19	Endrin	2.00E+00	5.00E+00	4.10E+01	1.80E+01	1.22E+01	0.00E+00	ECOTOX
20	Malachite green	4.00E+01	3.00E+00	0.00E+00	2.76E+00	1.29E+01	2.00E+00	ECOTOX
21	P-xylene	1.20E+04	1.00E+00	1.40E+01	0.00E+00	3.99E+00	0.00E+00	ECOTOX
22	Folpet	1.00E+02	2.00E+00	3.60E+01	5.97E+01	6.28E+00	6.00E+00	ECOTOX
23	Ethyl benzene	1.30E+04	1.00E+00	1.20E+01	0.00E+00	3.94E+00	0.00E+00	ECOTOX
24	Chlorothalonil	1.40E+02	1.00E+00	3.00E+01	3.32E+01	2.15E+00	6.00E+00	ECOTOX
25	Chlordane	4.70E+01	3.00E+00	3.70E+01	7.00E+00	1.04E+01	0.00E+00	ECOTOX
26	O-xylene	6.00E+03	1.00E+00	1.20E+01	0.00E+00	4.00E+00	0.00E+00	ECOTOX
27	Naphthalene	2.00E+03	2.00E+00	1.70E+01	0.00E+00	4.05E+00	0.00E+00	ECOTOX

*X_3__,1_ = nCIC, *X_3,2_* = UNIP, *X_3,3_* = P_VSA_LogP_4, *X_3,4_* = RDF015 m, and *X_3,5_* = CATS2D_04_AL; ** ECOTOX and PAN denote that the toxicity of the corresponding chemical is from the ECOTOX and PAN databases, respectively.

**Table 1d:** Values of the toxicities (LC_50_/EC_50_) and descriptors (*X*_*i,j*_)* of various chemicals to ten species, where *X*_*i,j*_ represents the *j*th descriptor in the *i*th QSAR model (*i* = 1,2,3, …,10; *j* = 1,2, …,8). Toxicities of 30 chemicals to *B. calyciflorus.*

No.	Chemical	LC_50_/EC_50_ (μg⋅L^−1^)	*X*_*4,1*_	*X*_*4,2*_	*X*_*4,3*_	*X*_*4,4*_	*X*_*4,5*_	*X*_*4,6*_	Resource**
1	Chlorpyrifos (CHL)	1.16E+04	3.51E+02	3.60E+02	1.19E+02	7.61E+00	9.79E+00	0.00E+00	ECOTOX
2	Paraquat (PAR)	1.94E+04	1.86E+02	1.68E+02	0.00E+00	8.20E+00	8.30E+00	0.00E+00	ECOTOX
3	Thiobencarb	1.78E+04	2.58E+02	2.58E+02	3.91E+01	7.12E+00	8.65E+00	0.00E+00	ECOTOX
4	Endothall	2.70E+05	1.86E+02	1.26E+02	0.00E+00	1.12E+01	1.01E+01	2.00E+00	ECOTOX
5	Benzene	3.16E+04	7.81E+01	0.00E+00	0.00E+00	1.42E+01	3.65E+00	0.00E+00	ECOTOX
6	Phenol	2.75E+05	9.41E+01	1.40E+01	0.00E+00	1.28E+01	3.92E+00	2.00E+00	ECOTOX
7	Toluene	1.13E+05	9.22E+01	1.40E+01	0.00E+00	1.32E+01	8.14E+00	0.00E+00	ECOTOX
8	Chloroform	7.81E+06	1.19E+02	6.00E+00	0.00E+00	3.05E+01	1.27E+01	0.00E+00	ECOTOX
9	Lindane	2.70E+03	2.91E+02	6.00E+01	0.00E+00	1.40E+01	1.14E+01	0.00E+00	ECOTOX
10	Nicotine	2.19E+05	1.62E+02	1.06E+02	0.00E+00	1.00E+01	8.42E+00	0.00E+00	ECOTOX
11	Methanol	3.59E+07	3.21E+01	0.00E+00	0.00E+00	3.40E+01	8.77E+00	0.00E+00	ECOTOX
12	Fenitrothion	6.69E+03	2.77E+02	2.98E+02	9.19E+01	8.12E+00	9.36E+00	0.00E+00	ECOTOX
13	1,1,1-trichloroethane1	6.42E+06	1.33E+02	1.20E+01	0.00E+00	2.68E+01	1.19E+01	0.00E+00	ECOTOX
14	2,4,6-trinitrotoluene	5.55E+03	2.27E+02	2.08E+02	1.52E+02	9.02E+00	1.08E+01	0.00E+00	ECOTOX
15	2,3,4,6-tetrachlorophenol	2.88E+03	2.32E+02	6.00E+01	1.57E+02	1.19E+01	5.37E+00	0.00E+00	ECOTOX
16	1-octanol	9.30E+04	1.30E+02	6.00E+01	0.00E+00	8.84E+00	8.33E+00	1.00E+00	ECOTOX
17	1,3,5-trinitrobenzene	1.40E+03	2.13E+02	1.68E+02	1.52E+02	8.66E+00	5.86E+00	0.00E+00	ECOTOX
18	Warfarin	4.44E+05	3.08E+02	7.12E+02	0.00E+00	6.02E+00	8.90E+00	2.00E+00	ECOTOX
19	Ethyl alcohol	2.97E+07	4.61E+01	2.00E+00	0.00E+00	2.57E+01	8.60E+00	1.00E+00	ECOTOX
20	Acetaminophen	5.31E+06	1.51E+02	9.00E+01	0.00E+00	1.17E+01	9.62E+00	5.00E+00	ECOTOX
21	3,4-dichloro aniline	6.17E+04	1.62E+02	3.30E+01	7.83E+01	1.13E+01	4.46E+00	2.00E+00	ECOTOX
22	2,4-dinitrochlorobenzene	1.30E+03	2.03E+02	1.29E+02	1.41E+02	9.80E+00	5.41E+00	0.00E+00	ECOTOX
23	2,4-D	1.93E+05	2.21E+02	1.55E+02	7.83E+01	1.01E+01	9.92E+00	0.00E+00	ECOTOX
24	Diazinon	2.92E+04	3.04E+02	4.33E+02	4.12E+01	5.43E+00	8.37E+00	0.00E+00	PAN
25	Methylene chloride	2.02E+06	8.49E+01	2.00E+00	0.00E+00	3.07E+01	1.11E+01	0.00E+00	PAN
26	Methyl parathion	1.62E+04	2.63E+02	2.58E+02	9.19E+01	9.18E+00	9.70E+00	0.00E+00	PAN
27	Acetylsalicylic acid	1.41E+05	1.80E+02	1.54E+02	0.00E+00	1.04E+01	1.01E+01	1.00E+00	PAN
28	Xylene	2.71E+05	1.06E+02	1.20E+01	0.00E+00	1.19E+01	8.13E+00	0.00E+00	PAN
29	PCP	2.11E+03	2.66E+02	6.00E+01	1.96E+02	1.16E+01	5.72E+00	0.00E+00	PAN
30	N-hexane	6.83E+04	8.62E+01	1.60E+01	0.00E+00	1.19E+01	8.09E+00	0.00E+00	PAN

*X_4__,1_ = MW, *X_4,2_* = CENT, *X_4,3_* = P_VSA_LogP_8, *X_4,4_* = HGM, *X_4,5_* = HATSe, and *X_4,6_* = CATS2D_02_DL; ** ECOTOX and PAN denote that the toxicity of the corresponding chemical is from the ECOTOX and PAN databases, respectively.

**Table 1e:** Values of the toxicities (LC_50_/EC_50_) and descriptors (*X*_*i,j*_)* of various chemicals to ten species, where *X*_*i,j*_ represents the *j*th descriptor in the *i*th QSAR model (*i* = 1,2,3, …,10; *j* = 1,2, …,8). Toxicities of 33 chemicals to *C. dubia.*

No.	Chemical	LC_50_/EC_50_ (μg⋅L^−1^)	*X*_*5,1*_	*X*_*5,2*_	*X*_*5,3*_	*X*_*5,4*_	*X*_*5,5*_	Resource**
1	Dimethoate (DIM)	1.55E+03	5.91E+00	−3.25E+00	0.00E+00	0.00E+00	0.00E+00	ECOTOX
2	Malathion (MAL)	2.00E+00	6.09E+00	−6.57E+00	2.00E+00	2.00E+00	4.00E+00	ECOTOX
3	Paraquat (PAR)	4.41E+02	5.51E+00	−2.02E+00	0.00E+00	2.00E+00	2.00E+00	ECOTOX
4	Chlorobenzene	1.27E+04	5.35E+00	−7.16E-01	0.00E+00	0.00E+00	0.00E+00	ECOTOX
5	Phenol	6.33E+03	5.10E+00	−4.20E-01	0.00E+00	2.00E+00	1.00E+00	ECOTOX
6	Chloroform	2.90E+05	4.28E+00	−8.35E-01	0.00E+00	0.00E+00	0.00E+00	ECOTOX
7	Nitrofen	2.17E+02	5.72E+00	−2.45E+00	1.00E+00	1.10E+01	4.00E+00	ECOTOX
8	*Para*-chlorophenol	9.00E+03	5.44E+00	−6.49E-01	0.00E+00	2.00E+00	0.00E+00	ECOTOX
9	Atrazine	3.00E+04	5.10E+00	−5.79E-01	0.00E+00	0.00E+00	0.00E+00	ECOTOX
10	Fipronil sulfone	1.85E+01	6.53E+00	−1.91E+00	0.00E+00	4.00E+00	5.00E+00	ECOTOX
11	Ethyl alcohol	6.21E+05	3.13E+00	−1.09E+00	0.00E+00	0.00E+00	0.00E+00	ECOTOX
12	Carbaryl	1.16E+01	5.75E+00	−2.99E+00	1.00E+00	3.00E+00	5.00E+00	ECOTOX
13	3,4,5-trichlorophenol	3.90E+02	5.81E+00	−1.39E+00	0.00E+00	0.00E+00	2.00E+00	ECOTOX
14	2,4-xylenol	4.02E+03	5.39E+00	−1.84E+00	0.00E+00	3.00E+00	1.00E+00	ECOTOX
15	Chlorfenvinphos	4.00E-01	6.11E+00	−5.70E+00	0.00E+00	2.00E+00	1.20E+01	ECOTOX
16	Carbofuran phenol	5.69E+01	5.47E+00	−3.47E+00	1.00E+00	4.00E+00	3.00E+00	ECOTOX
17	Carbofuran	2.60E+00	5.49E+00	−4.46E+00	2.00E+00	4.00E+00	4.00E+00	ECOTOX
18	Chromic acid	1.45E+02	6.08E+00	−4.52E-01	0.00E+00	0.00E+00	0.00E+00	ECOTOX
19	2,4,5-trichlorophenol	1.74E+03	5.77E+00	−1.28E+00	0.00E+00	2.00E+00	1.00E+00	ECOTOX
20	Esfenvalerate	2.20E-01	5.56E+00	−6.13E+00	2.00E+00	1.10E+01	1.40E+01	ECOTOX
21	Diazinon	5.16E-01	6.35E+00	−7.38E+00	1.00E+00	4.00E+00	9.00E+00	ECOTOX
22	Propanil	2.33E+03	5.68E+00	−3.01E+00	0.00E+00	1.00E+00	2.00E+00	ECOTOX
23	Methoxychlor	1.41E+01	5.96E+00	−4.54E+00	2.00E+00	4.00E+00	2.00E+00	ECOTOX
24	Fenthion	1.71E+00	6.35E+00	−3.43E+00	1.00E+00	2.00E+00	5.00E+00	ECOTOX
25	Propylene glycol	5.89E+05	3.95E+00	−1.38E+00	0.00E+00	1.00E+00	0.00E+00	ECOTOX
26	Methyl parathion	2.61E+00	6.37E+00	−1.87E+00	1.00E+00	8.00E+00	8.00E+00	PAN
27	PCP	3.26E+02	6.03E+00	−1.73E+00	0.00E+00	2.00E+00	2.00E+00	PAN
28	Parathion	2.30E-01	6.37E+00	−4.78E+00	1.00E+00	8.00E+00	1.20E+01	PAN
29	Molinate	5.00E+03	4.98E+00	−4.04E+00	0.00E+00	2.00E+00	4.00E+00	PAN
30	Mevinphos	9.50E-01	5.79E+00	−1.57E+00	1.00E+00	0.00E+00	8.00E+00	PAN
31	Butachlor	3.00E+03	5.53E+00	−1.17E+01	0.00E+00	7.00E+00	9.00E+00	PAN
32	Fluoranthene	4.50E+01	6.07E+00	−2.49E+00	0.00E+00	0.00E+00	0.00E+00	PAN
33	Alachlor	1.07E+04	5.53E+00	−8.77E+00	0.00E+00	6.00E+00	7.00E+00	PAN

*X_5__,1_ = SpDiam_B(m), *X_5,2_* = Mor03u, *X_5,3_* = O-060, *X_5,4_* = CATS2D_03_AL, and *X_5,5_* = CATS2D_04_AL; ** ECOTOX and PAN denote that the toxicity of the corresponding chemical is from the ECOTOX and PAN databases, respectively.

**Table 1f:** Values of the toxicities (LC_50_/EC_50_) and descriptors (*X*_*i,j*_)* of various chemicals to ten species, where *X*_*i,j*_ represents the *j*th descriptor in the *i*th QSAR model (*i* = 1,2,3, …,10; *j* = 1,2, …,8). Toxicities of 20 chemicals to *T. tubifex.*

No.	Chemical	LC_50_/EC_50_ (μg⋅L^−1^)	*X*_*6,1*_	*X*_*6,2*_	*X*_*6,3*_	*X*_*6,4*_	Resource**
1	Dimethoate (DIM)	3.80E+03	3.74E+00	3.82E+00	1.75E+00	1.58E+00	ECOTOX
2	Endosulfan ii (beta)	1.00E+03	3.39E+00	4.32E+00	2.82E+00	3.77E+00	ECOTOX
3	Dinitro cresol	5.80E+03	2.83E+00	3.44E+00	5.14E-01	5.77E+00	ECOTOX
4	Zineb	2.70E+02	3.16E+00	9.25E-01	3.39E+00	−7.02E-01	ECOTOX
5	Phenol	7.51E+05	7.07E-01	2.63E+00	0.00E+00	−1.69E+00	ECOTOX
6	Tefluthrin	5.28E+01	4.69E+00	5.78E+00	3.91E+00	9.89E+00	ECOTOX
7	Trichlorfon	4.60E+03	4.12E+00	4.35E+00	0.00E+00	−2.73E+00	ECOTOX
8	Brodifacoum	1.00E+04	2.55E+00	6.10E+00	4.32E+00	−3.42E+00	ECOTOX
9	4,5,6,7,8,8-hexachloro-1,3,3a,4,7,7a-hexahydro-4,7-methanoisobenzofuran	1.00E+05	3.32E+00	2.41E+00	0.00E+00	−9.10E-01	ECOTOX
10	(z)-tributyl [(1-oxo-9-octadecenyl)oxy]stannane	2.00E+01	5.92E+00	5.70E+00	4.11E+00	7.80E+00	ECOTOX
11	Chlorpropham	3.80E+03	3.00E+00	4.16E+00	2.38E+00	3.19E+00	ECOTOX
12	Meturin	2.16E+04	2.74E+00	3.88E+00	1.11E+00	3.17E+00	ECOTOX
13	Methyl paraoxon	4.47E+03	3.61E+00	4.54E+00	2.77E+00	−1.67E+00	ECOTOX
14	Lead acetate	1.95E+04	3.54E+00	2.54E+00	0.00E+00	7.28E-01	ECOTOX
15	Heptachlor epoxide	1.00E+04	3.54E+00	2.54E+00	0.00E+00	7.28E-01	ECOTOX
16	Heptachlor	1.83E+03	3.39E+00	2.46E+00	0.00E+00	1.61E+00	ECOTOX
17	EPTC	1.85E+04	3.46E+00	4.41E+00	1.69E+00	3.00E+00	PAN
18	Tributyltin oxide	6.00E+00	5.57E+00	4.36E+00	4.40E+00	4.79E+00	PAN
19	Endrin	2.20E+04	3.61E+00	2.71E+00	4.03E-01	1.14E+00	PAN
20	Thiram	6.70E+02	3.74E+00	1.41E+00	2.18E+00	−5.57E-01	PAN

*X_6__,1_ = DBI, *X_6,2_* = MAXDP, *X_6,3_* = ATS7m, and *X_6,4_* = Mor13s; ** ECOTOX and PAN denote that the toxicity of the corresponding chemical is from the ECOTOX and PAN databases, respectively.

**Table 1g:** Values of the toxicities (LC_50_/EC_50_) and descriptors (*X*_*i,j*_)* of various chemicals to ten species, where *X*_*i,j*_ represents the *j*th descriptor in the *i*th QSAR model (*i* = 1,2,3, …,10; *j* = 1,2, …,8). Toxicities of 26 chemicals to *C. riparius.*

No.	Chemical	LC_50_/EC_50_ (μg⋅L^−1^)	*X*_*7,1*_	*X*_*7,2*_	*X*_*7,3*_	*X*_*7,4*_	*X*_*7,5*_	Resource**
1	Malathion (MAL)	2.69E+04	0.00E+00	1.88E+00	4.81E-01	6.00E+00	1.00E+00	ECOTOX
2	Endosulfan sulfate	1.00E+02	6.00E+00	1.17E+00	3.01E-01	2.00E+00	5.00E+00	ECOTOX
3	Endosulfan	1.00E+02	6.00E+00	1.08E+00	1.41E-01	2.00E+00	5.00E+00	ECOTOX
4	Dichlorodiphenyl ether	1.35E+03	2.00E+00	1.15E+00	2.48E-01	4.00E+00	7.00E+00	ECOTOX
5	Ethyl acetate	7.50E+04	0.00E+00	1.43E+00	2.05E-01	3.00E+00	0.00E+00	ECOTOX
6	Pyridine	2.69E+04	0.00E+00	1.03E+00	1.55E-01	0.00E+00	0.00E+00	ECOTOX
7	Phenol	5.00E+05	0.00E+00	1.10E+00	2.58E-01	2.00E+00	0.00E+00	ECOTOX
8	Toluene	4.70E+04	0.00E+00	1.02E+00	2.25E-01	0.00E+00	0.00E+00	ECOTOX
9	P-dichlorobenzene	1.20E+04	2.00E+00	1.83E+00	1.24E-01	0.00E+00	0.00E+00	ECOTOX
10	Aniline	1.75E+05	0.00E+00	1.04E+00	3.08E-01	0.00E+00	0.00E+00	ECOTOX
11	3,4-dichloroaniline	1.60E+03	2.00E+00	1.17E+00	2.47E-01	0.00E+00	0.00E+00	ECOTOX
12	O-cresol	3.40E+04	0.00E+00	1.46E+00	2.98E-01	2.00E+00	0.00E+00	ECOTOX
13	1,2,3-trichlorobenzene	1.70E+03	3.00E+00	1.63E+00	9.30E-02	0.00E+00	1.00E+00	ECOTOX
14	Butyl benzyl phthalate	1.34E+03	0.00E+00	1.01E+00	5.72E-01	6.00E+00	1.30E+01	ECOTOX
15	Trichloro ethylene	6.40E+04	3.00E+00	0.00E+00	3.10E-02	0.00E+00	0.00E+00	ECOTOX
16	1,1,2-trichloroethane	1.47E+04	3.00E+00	0.00E+00	7.00E-02	0.00E+00	0.00E+00	ECOTOX
17	N-propyl alcohol	2.35E+04	0.00E+00	9.21E-01	2.44E-01	1.00E+00	0.00E+00	ECOTOX
18	Chloroform	8.43E+04	3.00E+00	0.00E+00	2.30E-02	0.00E+00	0.00E+00	ECOTOX
19	Dichlorvos	2.30E+04	2.00E+00	5.95E-01	1.72E-01	1.00E+00	0.00E+00	ECOTOX
20	Isopropanol	1.25E+07	0.00E+00	0.00E+00	2.44E-01	2.00E+00	0.00E+00	ECOTOX
21	Brestan	5.00E+01	0.00E+00	2.49E+00	5.40E-01	2.00E+00	1.20E+01	ECOTOX
22	Pentachlorobenzene	2.71E+02	5.00E+00	1.25E+00	3.10E-02	0.00E+00	4.00E+00	ECOTOX
23	Diazinon	1.57E+02	0.00E+00	1.67E+00	3.89E+00	3.00E+00	2.00E+00	PAN
24	Pentachlorophenol	3.13E+02	5.00E+00	1.18E+00	1.04E-01	2.00E+00	4.00E+00	PAN
25	Tributyltin oxide	1.10E+01	0.00E+00	2.87E+00	1.28E+00	6.00E+00	1.70E+01	PAN
26	Acetone	1.30E+07	0.00E+00	0.00E+00	1.58E-01	2.00E+00	0.00E+00	PAN

*X_7__,1_ = nCL, *X_7,2_* = GATS4m, *X_7,3_* = RDF010 m, *X_7,4_* = F02[C–O], and *X_7,5_* = CATS3D_05_LL; ** ECOTOX and PAN denote that the toxicity of the corresponding chemical is from the ECOTOX and PAN databases, respectively.

**Table 1h:** Values of the toxicities (LC_50_/EC_50_) and descriptors (*X*_*i,j*_)* of various chemicals to ten species, where *X*_*i,j*_ represents the *j*th descriptor in the *i*th QSAR model (*i* = 1,2,3, …,10; *j* = 1,2, …,8). Toxicities of 30 chemicals to *C. dipterum.*

No.	Chemical	LC_50_/EC_50_ (μg⋅L^−1^)	*X*_*8,1*_	*X*_*8,2*_	*X*_*8,3*_	*X*_*8,4*_	*X*_*8,5*_	Resource**
1	Chlorpyrifos (CHL)	1.00E+00	3.51E+02	7.11E+00	4.95E+00	4.38E+00	6.00E+00	ECOTOX
2	Paraquat (PAR)	2.80E+04	1.86E+02	5.00E+00	3.32E+00	1.40E+00	0.00E+00	ECOTOX
3	Thiobencarb	1.20E+3	2.58E+02	1.04E+01	5.96E+00	2.49E+00	2.00E+00	ECOTOX
4	2-propen-1-amine	3.00E+04	5.71E+01	3.84E+00	4.32E+00	7.18E-01	0.00E+00	ECOTOX
5	1-heptanol	1.34E+05	1.16E+02	0.00E+00	3.75E+00	7.39E-01	0.00E+00	ECOTOX
6	Propoxur	1.30E+02	2.09E+02	1.29E+01	5.38E+00	3.28E+00	0.00E+00	ECOTOX
7	Zineb	4.00E+04	2.10E+02	6.31E+00	3.27E+00	1.24E+00	0.00E+00	ECOTOX
8	Benzene	3.40E+04	7.81E+01	0.00E+00	7.63E-01	2.04E+00	0.00E+00	ECOTOX
9	Ethyl acetate	4.80E+05	8.81E+01	2.84E+00	2.28E+00	7.01E-01	0.00E+00	ECOTOX
10	Pyridine	1.65E+05	7.91E+01	0.00E+00	1.31E+00	1.60E+00	0.00E+00	ECOTOX
11	Phenol	3.44E+04	9.41E+01	1.16E+00	1.01E+00	1.89E+00	0.00E+00	ECOTOX
12	Aniline	2.20E+05	9.31E+01	2.50E+00	2.66E+00	1.87E+00	0.00E+00	ECOTOX
13	Salicylaldehyde	1.30E+04	1.22E+02	1.86E+00	2.03E+00	2.43E+00	0.00E+00	ECOTOX
14	Propanoic acid, ethyl ester	1.94E+05	1.02E+02	3.51E+00	2.54E+00	1.17E+00	0.00E+00	ECOTOX
15	O-cresol	5.00E+04	1.08E+02	1.85E+00	1.38E+00	2.26E+00	0.00E+00	ECOTOX
16	Trichloro ethylene	4.20E+04	1.31E+02	1.75E+00	2.74E-01	1.16E+00	0.00E+00	ECOTOX
17	Nitrofen	1.00E+04	2.84E+02	1.41E+01	9.34E-01	1.71E+00	0.00E+00	ECOTOX
18	Kasugamycin	4.00E+04	3.61E+02	8.58E+00	8.06E+00	1.79E+00	0.00E+00	ECOTOX
19	Ferbam	5.60E+02	2.77E+02	1.10E+01	9.57E+00	2.14E+00	2.20E+01	ECOTOX
20	Fenitrothion	3.20E+00	2.26E+02	1.34E+01	5.03E+00	1.99E+00	0.00E+00	ECOTOX
21	DDT	8.50E+02	2.37E+02	7.68E+00	6.57E+00	3.99E+00	1.60E+01	ECOTOX
22	Cartap	7.50E+01	2.01E+02	6.08E+00	2.87E+00	2.38E+00	0.00E+00	ECOTOX
23	Carbaryl	3.70E+02	3.04E+02	1.31E+01	6.53E+00	6.52E+00	6.00E+00	ECOTOX
24	PCP	5.90E+03	5.81E+01	1.30E+00	7.91E-01	6.85E-01	0.00E+00	ECOTOX
25	Acetone	7.60E+06	2.91E+02	9.79E+00	8.06E+00	1.94E+00	2.20E+01	PAN
26	Parathion	2.20E+00	2.66E+02	4.38E+00	2.00E-03	2.88E+00	0.00E+00	PAN
27	Chlorothalonil	1.80E+03	2.02E+02	3.07E+01	4.00E+00	1.63E+00	0.00E+00	PAN
28	Rotenone	5.60E+01	3.94E+02	3.79E+01	8.70E+00	2.64E+00	0.00E+00	PAN
29	Azinphos-methyl	1.23E+01	3.17E+02	1.33E+01	5.98E+00	2.26E+00	1.80E+01	PAN
30	Thiram	1.02E+03	2.40E+02	6.18E+00	5.71E+00	1.71E+00	0.00E+00	PAN

*X_8__,1_ = MW, *X_8,2_* = Chi1_EA(dm), *X_8,3_* = RDF020u, *X_8,4_* = L2u, and *X_8,5_* = CATS3D_04_AL; ** ECOTOX and PAN denote that the toxicity of the corresponding chemical is from the ECOTOX and PAN databases, respectively.

**Table 1i:** Values of the toxicities (LC_50_/EC_50_) and descriptors (*X*_*i,j*_)* of various chemicals to ten species, where *X*_*i,j*_ represents the *j*th descriptor in the *i*th QSAR model (*i* = 1,2,3, …,10; *j* = 1,2, …,8). Toxicities of 41 chemicals to *C. variegatus*.

No.	Chemical	LC_50_/EC_50_ (μg⋅L^−1^**)**	*X*_*9,1*_	*X*_*9,2*_	*X*_*9,3*_	*X*_*9,4*_	*X*_*9,5*_	*X*_*9,6*_	Resource**
1	Chlorpyrifos (CHL)	1.36E+02	3.51E+02	3.10E+01	4.24E+02	1.63E+00	0.00E+00	1.03E+01	ECOTOX
2	Glyphosate (GLY)	2.40E+05	1.69E+02	1.67E+01	2.12E+02	5.00E-01	4.58E-01	3.84E+00	ECOTOX
3	Dimethoate (DIM)	1.11E+05	2.29E+02	2.08E+01	2.44E+02	5.00E-01	6.07E-01	5.71E-01	ECOTOX
4	Acetochlor (ACE)	2.86E+03	2.70E+02	3.68E+01	2.56E+02	1.69E+00	1.10E+00	1.01E+01	ECOTOX
5	Dichloroethane	1.80E+05	9.90E+01	2.50E+01	1.08E+02	0.00E+00	0.00E+00	3.30E+00	ECOTOX
6	Methyl chloroform	7.10E+04	1.33E+02	2.50E+01	1.64E+02	0.00E+00	0.00E+00	4.96E+00	ECOTOX
7	Chlorobenzene	1.00E+04	1.13E+02	5.00E+01	1.10E+02	1.87E-01	0.00E+00	8.27E+00	ECOTOX
8	Diethanolamine	5.40E+05	1.05E+02	2.22E+01	8.20E+01	1.25E-01	0.00E+00	8.40E-01	ECOTOX
9	Diphenyl ether	2.40E+03	1.70E+02	5.22E+01	1.58E+02	3.75E-01	0.00E+00	1.15E+01	ECOTOX
10	Toluene	1.30E+04	9.22E+01	4.67E+01	6.20E+01	1.87E-01	0.00E+00	6.80E+00	ECOTOX
11	Camphorene	1.90E+03	2.61E+02	3.73E+01	1.22E+02	7.50E-01	6.80E-01	3.02E+01	ECOTOX
12	Tetrachloroethane	1.20E+04	1.68E+02	2.50E+01	2.16E+02	0.00E+00	0.00E+00	6.78E+00	ECOTOX
13	Acenaphthene	2.61E+03	1.54E+02	5.45E+01	1.26E+02	8.47E-01	0.00E+00	1.57E+01	ECOTOX
14	Trichloro ethylene	7.18E+04	1.31E+02	3.33E+01	1.72E+02	0.00E+00	0.00E+00	4.33E+00	ECOTOX
15	Dichlorvos	1.03E+04	2.21E+02	2.22E+01	2.94E+02	5.00E-01	0.00E+00	1.55E+00	ECOTOX
16	Nitrobenzene	5.90E+04	1.23E+02	4.29E+01	1.58E+02	4.37E-01	0.00E+00	3.56E+00	ECOTOX
17	Pentachloroethane	1.16E+05	2.02E+02	2.50E+01	2.70E+02	0.00E+00	1.20E+00	8.75E+00	ECOTOX
18	Cacodylic acid	9.90E+05	1.38E+02	1.67E+01	8.80E+01	0.00E+00	0.00E+00	1.44E+00	ECOTOX
19	Amitrole	1.00E+06	8.41E+01	2.00E+01	1.00E+02	2.22E-01	0.00E+00	5.40E-02	ECOTOX
20	Trichlorfon	1.53E+04	2.57E+02	2.00E+01	3.32E+02	1.50E+00	0.00E+00	2.39E-01	ECOTOX
21	Carbaryl	2.65E+03	2.01E+02	4.62E+01	2.16E+02	8.12E-01	3.66E-01	4.94E+00	ECOTOX
22	2,4-D	1.28E+04	2.21E+02	4.21E+01	2.90E+02	6.87E-01	9.22E-01	5.54E+00	ECOTOX
23	1-naphthol	1.80E+03	1.44E+02	5.26E+01	1.36E+02	6.87E-01	0.00E+00	6.95E+00	ECOTOX
24	Bronopol	5.90E+04	2.00E+02	2.00E+01	2.20E+02	6.25E-01	4.15E-01	4.20E-02	ECOTOX
25	1,2-dichlorobenzene	9.70E+03	1.47E+02	5.00E+01	1.66E+02	3.75E-01	0.00E+00	1.21E+01	ECOTOX
26	1,1-dichloroethylene	2.50E+05	9.69E+01	3.33E+01	1.18E+02	0.00E+00	0.00E+00	2.80E+00	ECOTOX
27	Bromoform	1.13E+04	2.53E+02	2.00E+01	1.56E+02	0.00E+00	−1.48E+00	5.85E+00	ECOTOX
28	Dichloromethane	3.30E+05	8.49E+01	2.00E+01	1.02E+02	0.00E+00	−1.20E+00	1.86E+00	ECOTOX
29	Fentin hydroxide	2.94E+01	3.50E+02	5.29E+01	1.92E+02	1.31E+00	0.00E+00	3.56E+01	ECOTOX
30	Hexachlorocyclopentadiene	4.50E+01	2.73E+02	4.55E+01	3.74E+02	2.21E+00	7.45E-01	1.27E+01	ECOTOX
31	2-butanone	4.00E+05	7.21E+01	3.08E+01	5.80E+01	0.00E+00	0.00E+00	4.29E-01	ECOTOX
32	Isophorone	1.40E+05	1.38E+02	3.75E+01	1.04E+02	1.38E+00	6.80E-01	3.79E+00	ECOTOX
33	DEF	7.67E+02	3.15E+02	2.73E+01	2.08E+02	7.50E-01	0.00E+00	1.10E+01	ECOTOX
34	Heptachlor	6.20E+00	3.73E+02	4.55E+01	4.68E+02	5.19E+00	1.46E+00	2.63E+01	ECOTOX
35	Butyl benzyl phthalate	9.65E+03	3.12E+02	4.42E+01	3.22E+02	1.31E+00	1.67E+00	1.67E+01	ECOTOX
36	10,10-oxybisphenoxyarsine	8.00E+00	5.02E+02	5.33E+01	4.30E+02	1.88E+00	8.60E-01	1.25E+01	ECOTOX
37	Endrin	4.00E-01	3.81E+02	4.44E+01	4.68E+02	4.94E+00	1.46E+00	1.98E+01	PAN
38	Fenthion	1.20E+03	2.78E+02	3.23E+01	2.84E+02	1.31E+00	0.00E+00	6.21E+00	PAN
39	Captan	1.90E+03	3.01E+02	3.75E+01	3.72E+02	2.36E+00	2.00E+00	3.32E+00	PAN
40	Dimethyl phthalate	4.10E+04	1.94E+02	4.17E+01	2.46E+02	1.13E+00	1.67E+00	4.02E+00	PAN
41	Diethyl phthalate	2.95E+04	2.22E+02	4.00E+01	2.54E+02	1.38E+00	1.67E+00	6.64E+00	PAN

*X_9__,1_ = MW, *X_9,2_* = C%, *X_9,3_* = ZM1V, *X_9,4_* = GGI3, *X_9,5_* = Eig02_EA(dm), and *X_9,6_* = MLOGP2; ** ECOTOX and PAN denote that the toxicity of the corresponding chemical is from the ECOTOX and PAN databases, respectively.

**Table 1j:** Values of the toxicities (LC_50_/EC_50_) and descriptors (*X*_*i,j*_)* of various chemicals to ten species, where *X*_*i,j*_ represents the *j*th descriptor in the *i*th QSAR model (*i* = 1,2,3, …,10; *j* = 1,2, …,8). Toxicities of 39 chemicals to *S. capricornutum.*

No.	Chemical	LC_50_/EC_50_ (μg⋅L^−1^)	*X*_*10,1*_	*X*_*10,2*_	*X*_*10,3*_	*X*_*10,4*_	*X*_*10,5*_	*X*_*10,6*_	*X*_*10,7*_	Resource**
1	Chlorpyrifos (CHL)	7.69E+02	3.40E+00	2.74E+01	1.37E+00	4.23E+01	4.70E+01	0.00E+00	0.00E+00	ECOTOX
2	Acetochlor (ACE)	1.43E+03	3.30E+00	2.12E+01	9.86E-01	4.08E+01	2.35E+01	0.00E+00	0.00E+00	ECOTOX
3	Cyanazine	2.00E+02	3.22E+00	1.78E+01	1.85E+00	1.03E+02	4.56E+01	0.00E+00	3.00E+00	ECOTOX
4	Mefenacet	2.00E+02	4.03E+00	1.90E+01	1.58E+00	4.66E+01	7.46E-01	1.00E+00	0.00E+00	ECOTOX
5	Cyclosulfamuron	1.00E+02	4.47E+00	3.16E+01	1.53E+00	4.89E+01	9.33E+01	0.00E+00	0.00E+00	ECOTOX
6	3-chloro-p-toluidine hydrochloride	1.60E+04	6.93E-01	1.39E+01	9.12E-01	1.20E+01	4.45E+01	0.00E+00	2.00E+00	ECOTOX
7	Methyl chloroform	5.00E+05	0.00E+00	0.00E+00	0.00E+00	0.00E+00	1.17E+02	0.00E+00	0.00E+00	ECOTOX
8	3,4-dichloroaniline	2.03E+03	6.93E-01	1.01E+01	1.39E+00	0.00E+00	0.00E+00	0.00E+00	2.00E+00	ECOTOX
9	Trichloro ethylene	4.19E+05	0.00E+00	0.00E+00	0.00E+00	0.00E+00	9.01E+01	0.00E+00	0.00E+00	ECOTOX
10	1,1,2-trichloroethane	2.07E+05	0.00E+00	0.00E+00	0.00E+00	0.00E+00	1.18E+01	0.00E+00	0.00E+00	ECOTOX
11	Lindane	1.62E+03	1.95E+00	5.53E+01	1.25E+00	0.00E+00	0.00E+00	0.00E+00	0.00E+00	ECOTOX
12	1-chloronaphthalene	8.50E+02	3.05E+00	9.54E+00	1.75E+00	0.00E+00	0.00E+00	0.00E+00	0.00E+00	ECOTOX
13	1,3-dichloropropane	4.90E+04	0.00E+00	1.32E+01	0.00E+00	0.00E+00	0.00E+00	0.00E+00	0.00E+00	ECOTOX
14	1,3-dichlorobenzene	1.25E+04	0.00E+00	1.01E+01	2.61E+00	0.00E+00	0.00E+00	0.00E+00	0.00E+00	ECOTOX
15	1,1,2,2-tetrachloroethane	8.90E+04	0.00E+00	0.00E+00	0.00E+00	0.00E+00	2.35E+01	0.00E+00	0.00E+00	ECOTOX
16	1,2-benzenedicarboxylic acid, ditridecyl ester	6.00E+02	3.97E+00	5.28E+01	1.23E+00	6.47E+01	0.00E+00	0.00E+00	0.00E+00	ECOTOX
17	1,2-benzenedicarboxylic acid, diisononyl ester	1.80E+03	3.81E+00	4.28E+01	1.34E+00	6.47E+01	0.00E+00	0.00E+00	0.00E+00	ECOTOX
18	1,2,4-trichlorobenzene	9.46E+03	6.93E-01	1.44E+01	1.01E+00	0.00E+00	0.00E+00	0.00E+00	0.00E+00	ECOTOX
19	1,2,4,5-tetrachlorobenzene	4.98E+04	1.10E+00	1.68E+01	0.00E+00	0.00E+00	0.00E+00	0.00E+00	0.00E+00	ECOTOX
20	1,2,3,5-tetrachlorobenzene	1.74E+04	1.10E+00	1.68E+01	1.79E+00	0.00E+00	0.00E+00	0.00E+00	0.00E+00	ECOTOX
21	1,1,1-trichloroethane	5.00E+05	0.00E+00	0.00E+00	0.00E+00	0.00E+00	1.17E+02	0.00E+00	0.00E+00	ECOTOX
22	2,3,4-trichlorophenol	2.00E+03	1.39E+00	1.50E+01	1.24E+00	4.27E+01	0.00E+00	0.00E+00	2.00E+00	ECOTOX
23	2,3,4,6-tetrachlorophenol	1.30E+03	1.61E+00	1.80E+01	7.99E-01	4.27E+01	0.00E+00	0.00E+00	2.00E+00	ECOTOX
24	2,4-D	2.50E+04	2.49E+00	1.12E+01	8.03E-01	6.87E+01	0.00E+00	0.00E+00	0.00E+00	ECOTOX
25	Propylene dichloride	8.30E+04	0.00E+00	8.42E+00	0.00E+00	0.00E+00	0.00E+00	0.00E+00	0.00E+00	ECOTOX
26	Pentachlorophenol	2.69E+02	1.95E+00	1.81E+01	8.12E-01	4.27E+01	0.00E+00	0.00E+00	2.00E+00	ECOTOX
27	1,1-dichloroethylene	4.79E+05	0.00E+00	0.00E+00	0.00E+00	0.00E+00	7.83E+01	0.00E+00	0.00E+00	ECOTOX
28	Dichloromethane	5.00E+05	0.00E+00	0.00E+00	0.00E+00	0.00E+00	2.35E+01	0.00E+00	0.00E+00	ECOTOX
29	Methyl methacrylate	8.61E+04	0.00E+00	7.12E+00	9.90E-02	3.23E+01	0.00E+00	1.00E+00	0.00E+00	ECOTOX
30	Diethyl phthalate	1.02E+05	3.14E+00	1.23E+01	1.56E+00	6.47E+01	0.00E+00	0.00E+00	0.00E+00	ECOTOX
31	Buctril	2.90E+03	1.95E+00	6.39E+01	1.02E+00	5.93E+01	0.00E+00	0.00E+00	0.00E+00	ECOTOX
32	Tetrachloroethylene	5.00E+05	0.00E+00	0.00E+00	0.00E+00	0.00E+00	1.57E+02	0.00E+00	0.00E+00	PAN
33	Glufosinate	4.65E+04	0.00E+00	1.74E+01	9.30E-01	8.90E+01	0.00E+00	1.00E+00	1.00E+00	PAN
34	Flumetsulam	1.92E+04	4.32E+00	1.32E+01	1.05E+00	1.11E+02	4.24E+01	0.00E+00	0.00E+00	PAN
35	Ethametsulfuron	8.60E+03	4.14E+00	2.12E+01	1.34E+00	2.07E+02	1.25E+01	1.00E+00	1.00E+00	PAN
36	Prometryn	1.35E+01	3.37E+00	2.13E+01	2.42E+00	8.64E+01	4.49E+00	1.00E+00	4.00E+00	PAN
37	Metolachlor	2.06E+02	3.37E+00	2.55E+01	1.02E+00	4.08E+01	0.00E+00	1.00E+00	0.00E+00	PAN
38	Isoproturon	2.10E+01	3.14E+00	1.90E+01	8.72E-01	2.51E+01	4.28E+00	2.00E+00	2.00E+00	PAN
39	Metribuzin	8.08E+01	3.09E+00	2.40E+01	2.83E+00	3.26E+01	6.93E-01	1.00E+00	1.00E+00	PAN

*X_10__,1_ = MPC07, *X_10,2_* = ATSC4m, *X_10,3_* = GATS5e, *X_10,4_* = P_VSA_LogP_4, *X_10,5_* = P_VSA_LogP_6, *X_10,6_* = C-005, and *X_10,7_* = CATS3D_02_DL; ** ECOTOX and PAN denote that the toxicity of the corresponding chemical is from the ECOTOX and PAN databases, respectively.

**Table 2a:** Toxicities of six pesticides to various species. Acetochlor toxicity to 17 species.

No.	Species	LC_50_ (μg⋅L^−1^)	Resource*
1	*Xenopus laevis*	3.03E+03/2.42E+03	ECOTOX
2	*Bufo gargarizans tadpoles*	1.33E+03	ECOTOX
3	*Bufo gargarizans*	1.32E+03	ECOTOX
4	*Sheepshead minnow*	3.90E+03/2.10E+03	ECOTOX
5	*Bluegill*	1.5E+03/1.6E+03/1.3E+03	ECOTOX
6	*Rainbow trout, Donaldson trout*	1.20E+03/3.80E+02/4.20E+02	ECOTOX
7	*Opossum shrimp*	5.30E+03/2.20E+03	ECOTOX
8	*Danio rerio*	3.04E+03/7.03E+02	PAN
9	*Gobiocypris rarus*	8.27E+02	PAN
10	*B. b. japonicus*	**3.33E**+**03**	QSAR
11	*C. magister*	**5.00E**+**00**	QSAR
12	*T. tubifex*	**1.21E**+**03**	QSAR
13	*C. riparius*	**5.83E**+**03**	QSAR
14	*B. calyciflorus*	**4.63E**+**04**	QSAR
15	*C. dubia*	**8.72E**+**02**	QSAR
16	*C. dipterum*	**4.39E**+**00**	QSAR
17	*C. variegatus*	**3.35E**+**03**	QSAR

*ECOTOX, PAN, and QSAR denote that the toxicity of the chemical to the species is from the ECOTOX database, PAN database, and QSAR prediction, respectively. Bold: EC_50_/LC_50_ (μg·L^-1^) predicted by the developed QSAR models.

**Table 2b:** Toxicities of six pesticides to various species. Chlorpyrifos toxicity to 97 species.

No.	Species	LC_50_ (μg⋅L^−1^)	Resource*
1	*Clawed toad*	1.46E+04/5.64 E+02/2.41 E+03	ECOTOX
2	*Amphipod*	7.00E-02/1.40E-01/3.90E-01	ECOTOX
3	*Tiger frog*	1.90E+01	ECOTOX
4	*Oligochaete*	6.60E+01	ECOTOX
5	*Red swamp crayfish*	4.50 E+02/2.1 E+01	ECOTOX
6	*Threespine stickleback*	8.50E+00	ECOTOX
7	*Indian catfish*	2.20E+03	ECOTOX
8	*Channel catfish*	8.06 E+02/2.08 E+03	ECOTOX
9	*Rohu, Labeo rohita*	2.35E+03	ECOTOX
10	*Opossum shrimp*	1.50E-01	ECOTOX
11	*Shrimp*	2.80E-01/1.50E-01	ECOTOX
12	*Chinese Mitten Crab*	2.29E+01/1.44E+02/7.59E+01	ECOTOX
13	*Crayfish*	6.00E+00	ECOTOX
14	*Crab*	2.00E+02/6.00E+02	ECOTOX
15	*California grunion*	5.50E+00/6.00E+00/2.80E+00	ECOTOX
16	*Inland silverside*	1.03E+01/4.20E+00	ECOTOX
17	*Atlantic silverside*	5.00E-01/2.80E+00/4.40E+00/1.70E+00	ECOTOX
18	*Tidewater silverside*	1.03E+01/4.20E+00/2.00E+00	ECOTOX
19	*Korean or Oriental shrimp*	2.50E-01/1.00E-02	ECOTOX
20	*White river crayfish*	2.00E+00	ECOTOX
21	*Western mosquitofish*	5.70E+02	ECOTOX
22	*Bluegill*	3.00E+01/2.80E+02	ECOTOX
23	*Fathead minnow*	1.30E+02/1.70E+02	ECOTOX
24	*Striped bass*	5.80E-01/1.00E+03	ECOTOX
25	*Striped mullet*	5.40E+00	ECOTOX
26	*Cutthroat trout*	1.84E+01	ECOTOX
27	*Gulf toadfish*	5.20E+02	ECOTOX
28	*Nile tilapia(young)*	9.87E+01	ECOTOX
29	*Nile tilapia(adult)*	1.51E+02	ECOTOX
30	*Mummichog*	4.65E+00	ECOTOX
31	*Rainbow trout*	8.00E+00/4.50E+01	ECOTOX
32	*Guppy*	7.17E+00	ECOTOX
33	*Ninespine stickleback*	4.70E+00	ECOTOX
34	*Rutilus rutilus*	1.20E+02	ECOTOX
35	*Lake trout, siscowet*	1.40E+02	ECOTOX
36	*Tilapia*	2.40E+02	ECOTOX
37	*Longnose killifish*	4.10E+00	ECOTOX
38	*Common eel*	5.40E+02	ECOTOX
39	*Tooth carp*	1.70E-01	ECOTOX
40	*Topsmelt*	5.50E+00/4.50E+00	ECOTOX
41	*Silver perch*	1.70E+01	ECOTOX
42	*Mozambique tilapia*	2.60E+01	ECOTOX
43	*Rana dalmatina tadpoles*	5.17E+03	ECOTOX
44	*Heteropneustes fossilis*	1.76E+03/4.40E+02	ECOTOX
45	*The marine invertebrate Donax faba*	2.48E+02	ECOTOX
46	*Goldfish*	8.06E+02	ECOTOX
47	*Catla*	1.66E+03	ECOTOX
48	*Snake-head catfish*	3.65E+02	ECOTOX
49	*Carp*	2.35E+03/6.50E+02	ECOTOX
50	*Sheepshead minnow*	1.36E+02	ECOTOX
51	*Ten Spotted Live- Bearer Fis*	2.10E+02	ECOTOX
52	*h Adult riceland prawns*	2.53E+00	ECOTOX
53	*Common carp*	1.49E+02	ECOTOX
54	*C. Gariepinus*	8.60E+02	ECOTOX
55	*The freshwater fish Puntius chola*	2.19E+02	ECOTOX
56	*African catfish Clarias gariepinus*	8.61E+02	ECOTOX
57	*Rainbow trout*	9.00E+00	ECOTOX
58	*Labeo rohita*	4.43E+02	ECOTOX
59	*Chinese toad tadpoles*	8.00E+02	ECOTOX
60	*Adult zebrafish*	7.09E+02	ECOTOX
61	*Oryzias latipes*	2.67E+02	ECOTOX
62	*Mosquito fish, Gambusia affinis*	2.97E+02	ECOTOX
63	*Cyprinus carpio*	1.60E+02	ECOTOX
64	*Freshwater teleost fish Channa punctatus*	8.12E+02	ECOTOX
65	*Freshwater prawn Palaemonetes argentinus*	4.90E-01	ECOTOX
66	*Nile tilapia larvae*	1.57E+03	ECOTOX
67	*The fry of common carp*	8.00E+00	ECOTOX
68	*Fish Oreochromis mossambicus*	2.60E+01	ECOTOX
69	*Estuarine amphipod Gammarus palustris*	3.00E-01	ECOTOX
70	*Neomysis integer*	1.30E-01	ECOTOX
71	*Neomysis integer*	1.90E-01	ECOTOX
72	*Freshwater catfish*	2.20E+03	ECOTOX
73	*Grass shrimp*	3.70E-01	ECOTOX
74	*Grass shrimp*	4.40E-01	ECOTOX
75	*Anguilla anguilla*	5.40E-01	ECOTOX
76	*Pimephales promelas*	1.22E+02	ECOTOX
77	*Rhinella fernandezae*	1.51E+02	ECOTOX
78	*Mosquitofish (Gambusia yucatana)*	8.50E+01	ECOTOX
79	*Brachydanio rerio*	1.11E+03	ECOTOX
80	*Zebra fish*	1.94E+03	ECOTOX
81	*Xiphophorus*	1.71E+02	PAN
82	*Pseudorasbora parva*	2.73E+01	PAN
83	*Sunfish*	6.81E+01	PAN
84	*Odontobutis potamophila*	5.20E+01	PAN
85	*Rare minnow embryo*	7.59E+03	PAN
86	*Brachydanio rerio*	1.11E+03	PAN
87	*Carassius auratus*	1.85E+02	PAN
88	*The embryos of Microhyla pulchra*	1.97E+03	PAN
89	*Silver carp*	1.72E+02	PAN
90	*Litopenaeus vannamei*	7.58E-01	PAN
91	*Paramisgurnus dabryanus*	1.93E+02	PAN
92	*Chironomus riparius*	9.00E-02	PAN
93	*Chironomus plumosus*	1.30E+00/2.53E+02	PAN
94	*B. b. japonicus*	**1.27E**+**04**	QSAR
95	*C. magister*	**2.50E**+**01**	QSAR
96	*T. tubifex*	**1.76E**+**02**	QSAR
97	*C. riparius*	**1.72E** + **03**	QSAR

*ECOTOX, PAN, and QSAR denote that the toxicity of the chemical to the species is from the ECOTOX database, PAN database, and QSAR prediction, respectively. Bold: EC_50_/LC_50_ (μg·L^-1^) predicted by the developed QSAR models.

**Table 2c:** Toxicities of six pesticides to various species. Dimethoate toxicity to 56 species.

No.	Species	LC_50_ (μg⋅L^−1^)	Resource*
1	*Gammarus pulex*	2.20E+00	ECOTOX
2	*Cyprinus carpio*	2.61E+04	ECOTOX
3	*Lepomis macrochirus*	6.00E+03	ECOTOX
4	*Mugilidae*	2.30E+03	ECOTOX
5	*Rana cyanophlyctis*	3.60E+04	ECOTOX
6	*Clarias batrachus*	6.50E+03	ECOTOX
7	*Metapenaeus ensis*	2.31E+03	ECOTOX
8	*Daphnia magna*	5.04E+03/6.40E+03	ECOTOX
9	*Pelteobagrus fulvidraco*	7.55E+04	ECOTOX
10	*Asellus aquaticus*	2.96E+03	ECOTOX
11	*Fundlus similis*	1.00E+03	ECOTOX
12	*Lymnaea stagnalis*	1.92E+04	ECOTOX
13	*Brachydanio rerio*	3.16E+04	ECOTOX
14	*Pteronarcys californica*	4.30E+01	ECOTOX
15	*Aedes aegypti*	5.04E+03	ECOTOX
16	*Oncorhynchus mykiss*	6.20E+03/7.50E+03	ECOTOX
17	*Rana hexadactyla*	7.82E+00	ECOTOX
18	*Bufo Melanostictus Schneider*	1.32E+05	ECOTOX
19	*Channa punctata*	2.05E+04	ECOTOX
20	*Catla catla*	1.05E+04	ECOTOX
21	*Cyclops strenuus*	2.00E+03	ECOTOX
22	*Tooth carp*	1.17E+02	ECOTOX
23	*Echinogammarus tibaldii*	4.10E+03	ECOTOX
24	*Rosy barb*	4.78E+03	ECOTOX
25	*Cirrhinus mrigala*	1.01E+04	ECOTOX
26	*Channa orientalis*	4.48E+03	ECOTOX
27	*Clarias batrachus*	5.00E+03	ECOTOX
28	*Cyprinodon variegatus*	1.11E+05	ECOTOX
29	*Cyprinus carpio*	2.61E+04/4.65E+03	ECOTOX
30	*Danio rerio*	6.80E+03/7.80E+03	ECOTOX
31	*Lepomis macrochirus*	6.00E+03/9.60E+03	ECOTOX
32	*Indian catfish*	3.90E+04/1.05E+04/4.57E+03	ECOTOX
33	*Labeo rohita*	1.02E+04	ECOTOX
34	*Catfish*	2.20E+04	ECOTOX
35	*Mugilidae*	2.30E+00	ECOTOX
36	*Phoxinus phoxinus*	5.00E+02	ECOTOX
37	*Rutilus rutilus*	5.00E+02	ECOTOX
38	*Salmo salar*	1.30E+02	ECOTOX
39	*Salmo trutta*	1.30E+02/1.40E+02	ECOTOX
40	*Salvelinus alpinus*	1.30E+02	ECOTOX
41	*Salvelinus namaycush*	1.30E+02	ECOTOX
42	*Therapon jarbua*	7.00E+02	PAN
43	*Sheepshead minnow*	1.30E+04	PAN
44	*Tilapia nilotica*	1.11E+05	PAN
45	*Rainbow trout*	8.60E+03/6.20E+03/7.50E+03	PAN
46	*Rana cyanophlyctis*	3.60E+04/3.90E+04	PAN
47	*Guppy*	1.83E+04/1.30E+04/5.60E+05	PAN
48	*Cyclops strenuus*	2.00E+03	PAN
49	*Lepomis macrochirus*	6.00E+03	PAN
50	*X. laevis*	**8.51E**+**03**	QSAR
51	*B. b. japonicus*	**2.64E**+**05**	QSAR
52	*C. magister*	**4.19E**+**04**	QSAR
53	*B. calyciflorus*	**2.06E**+**04**	QSAR
54	*T. tubifex*	**2.67E**+**03**	QSAR
55	*C. riparius*	**1.01E**+**04**	QSAR
56	*C. dipterum*	**5.60E**+**02**	QSAR

*ECOTOX, PAN, and QSAR denote that the toxicity of the chemical to the species is from the ECOTOX database, PAN database, and QSAR prediction, respectively. Bold: EC_50_/LC_50_ (μg·L^-1^) predicted by the developed QSAR models.

**Table 2d:** Toxicities of six pesticides to various species. Glyphosate toxicity to 40 species.

No.	Species	LC_50_ (μg⋅L^−1^)	Resource*
1	*Piaractus mesopotamicus*	8.92E+03	ECOTOX
2	*Poecilia reticulata*	6.88E+04/7.09E+04	ECOTOX
3	*E.sinensis*	9.79E+04	ECOTOX
4	*Crayfish*	7.00E+03/2.16E+04	ECOTOX
5	*Rainbow trout*	1.00E+04	ECOTOX
6	*Goldfish*	2.56E+04	ECOTOX
7	*Sheepshead minnow*	2.40E+05	ECOTOX
8	*Cyprinus carpio*	1.10E+04	ECOTOX
9	*Channel catfish*	1.30E+04/4.40E+03	ECOTOX
10	*Bluegill*	5.00E+03/1.30E+05	ECOTOX
11	*Pink salmon*	1.40E+04	ECOTOX
12	*Chum salmon*	1.00E+04	ECOTOX
13	*Coho salmon*	2.70E+04	ECOTOX
14	*Chinook salmon*	1.90E+04	ECOTOX
15	*Fathead minnow*	9.70E+04/2.30E+03	ECOTOX
16	*Brown trout*	5.40E+03	ECOTOX
17	*Water flea*	1.75E+03/3.29E+03	ECOTOX
18	*Opossum shrimp*	4.00E+04/7.90E+04	ECOTOX
19	*Gammarus pseudolimnaeus*	4.30E+04/2.10E+05	ECOTOX
20	*American toad*	1.29E+04/2.58E+04	ECOTOX
21	*Frog*	7.80E+04/3.97E+04	ECOTOX
22	*Green frog*	6.50E+03/2.28E+04	ECOTOX
23	*Leopard frog*	9.20E+03/2.09E+04	ECOTOX
24	*Wood frog*	1.65E+04/2.58E+04	ECOTOX
25	*Clawed toad*	1.18E+04/6.90E+03	ECOTOX
26	*Leech*	1.18E+03/2.01E+02	ECOTOX
27	*Red swamp crayfish*	4.73E+04	ECOTOX
28	*Fiddler crab*	9.34E+05	ECOTOX
29	*Alburnus alburnus*	1.60E+04	ECOTOX
30	*Ten-spotted livebearer*	1.00E+05	ECOTOX
31	*Grass carp*	1.50E+04	ECOTOX
32	*Cyprinus carpio*	3.10E+03	PAN
33	*Striped bass*	2.35E+04	PAN
34	*Sockeye salmon*	2.67E+04	PAN
35	*American or virginia oyster*	1.00E+04	PAN
36	*B. b. japonicus*	**4.66E**+**04**	QSAR
37	*C. magister*	**2.42E**+**05**	QSAR
38	*T. tubifex*	**1.51E**+**04**	QSAR
39	*C. riparius*	**3.95E**+**05**	QSAR
40	*C. dipterum*	**1.92E**+**04**	QSAR

*ECOTOX, PAN, and QSAR denote that the toxicity of the chemical on the species is from the ECOTOX database, PAN database, and QSAR prediction, respectively. Bold: EC_50_/LC_50_ (μg·L^-1^) predicted by the developed QSAR models.

**Table 2e:** Toxicities of six pesticides to various species. Malathion toxicity to 29 species.

No.	Species	LC_50_ (μg⋅L^−1^)	Resource*
1	*Alburnus*	3.78E+03	ECOTOX
2	*Barbus dorsalis*	3.70E+03/3.20E+03	ECOTOX
3	*Barytelphusa cunicularis*	3.78E+03	ECOTOX
4	*Bufo tadpoles*	1.81E+03	ECOTOX
5	*Bufo woodhousei*	4.20E+02/1.90E+03/5.00E+02	ECOTOX
6	*Channel catfish*	9.65E+03/5.22E+04/8.97E+03	ECOTOX
7	*Chironomus plumosus*	8.40E+00/2.62E+02	ECOTOX
8	*Cirrhinus mrigala*	2.25E+03/8.80E+02/1.50E+01	ECOTOX
9	*Clarias batrachus*	2.50E+02	ECOTOX
10	Clawed toad	1.09E+04/9.00E+02	ECOTOX
11	*Cyprinella lutrensis*	2.50E+01	ECOTOX
12	*Cyprinus carpio*	2.00E+00/1.02E+04	ECOTOX
13	*Danio rerio*	1.05E+03	ECOTOX
14	*Fathead minnow*	1.80E+04	ECOTOX
15	*Frog and toad order*	1.50E+03	ECOTOX
16	*Giant river prawn*	9.00E+00/1.30E+01	ECOTOX
17	*Guppy*	1.20E+03	ECOTOX
18	*Indian catfish*	8.50E+03/1.50E+04	ECOTOX
19	*Lepomis macrochirus*	5.50E+02/9.00E+01	ECOTOX
20	*Northern chorus frog*	2.00E+02/5.60E+02	ECOTOX
21	*Oncorhynchus mykiss*	2.50E+02	ECOTOX
22	*Opposum Shrimp*	3.80E+00/1.40E+00/2.20E+00/1.50E+00	ECOTOX
23	*Procambarus clarkii*	1.34E+03/1.75E+03/4.92E+04	PAN
24	*Rainbow trout*	1.77E+02	PAN
25	*Sheepshead minnow*	3.30E+01/5.50E+01/5.10E+01	PAN
26	*White shrimp*	2.15E+01	PAN
27	*B. b. japonicus*	**9.86E**+**03**	QSAR
28	*C. dipterum*	**3.07E-02**	QSAR
29	*S. capricornutum*	**4.19E**+**02**	QSAR

*ECOTOX, PAN, and QSAR denote that the toxicity of the chemical to the species is from the ECOTOX database, PAN database, and QSAR prediction, respectively. Bold: EC_50_/LC_50_ (μg·L^-1^) predicted by the developed QSAR models.

**Table 2f:** Toxicities of six pesticides to various species. Paraquat toxicity to 30 species.

No.	Species	LC_50_ (μg⋅L^−1^)	Resource*
1	*Xenopus laevis*	8.10E+03	ECOTOX
2	*Bufo woodhousei fowleri*	1.50E+04	ECOTOX
3	*Striped, Northern chorus frog*	2.80E+04	ECOTOX
4	*Western chorus frog*	2.80E+04	ECOTOX
5	*Leopard frog, Rana pipiens*	1300/500	ECOTOX
6	*Frog, Scinax nasica*	2.20E+04	ECOTOX
7	*Cyprinus carpio*	1.50E+04	ECOTOX
8	*Rainbow trout, donaldson trout*	1.50E+04/2.90E+04/3.87E+04	ECOTOX
9	*Characin, Bryconamericus boops*	2.02E+04	ECOTOX
10	*Cnesterodon decemmaculatus*	9.41E+03	ECOTOX
11	*Danio rerio*	4.00E+04	ECOTOX
12	*Bluegill*, *Lepomis macrochirus*	1.30E+04/1.56E+04	ECOTOX
13	*Tilapia, Tilapia hornorum*	3.15E+04	ECOTOX
14	*Gammarus fasciatus*	1.10E+04	ECOTOX
15	*Gammarus lacustris*	1.10E+04	ECOTOX
16	*Physa acuta adult*	1.41E+03	ECOTOX
17	*Physa acuta embryo*	1.63E+03	ECOTOX
18	*Hydra*	8.09E+03	ECOTOX
19	*Bufo gargargarizans tadpoles*	2.01E+04	ECOTOX
20	*Carp*	1.51E+04	ECOTOX
21	*Polypedates megacephalus tadpoles*	1.21E+03	ECOTOX
22	*Black-orbited toad tadpoles*	2.04E+03	ECOTOX
23	*Juveniles of the African catfish*	7.00E+01	ECOTOX
24	*Colossoma macropomum*	4.81E+04	ECOTOX
25	*Rainbow trout*	3.58E+01	PAN
26	*Opposum Shrimp*	1.18E+04	PAN
27	*Green alga chlorella*	1.00E-01	PAN
28	*Grass carp*	1.30E+04	PAN
29	*C. magister*	**1.56E**+**03**	QSAR
30	*C. riparius*	**6.42E**+**04**	QSAR

*ECOTOX, PAN, and QSAR denote that the toxicity of the chemical to the species is from the ECOTOX database, PAN database, and QSAR prediction, respectively. Bold: EC_50_/LC_50_ (μg·L^-1^) predicted by the developed QSAR models.

Table 1a, Table 1b, Table 1c, Table 1d, Table 1e, Table 1f, Table 1g, Table 1h, Table 1i, Table 1j lists the toxicity values (EC_50_ or LC_50_ (μg⋅L^−1^)) of various chemicals for ten different species and the values of the descriptors input in the ten QSAR models. The ten species include two amphibians (*Xenopus laevis* (*X. laevis*) and *Bufo bufo japonicus* (*B. b. japonicus*)), two crustaceans (*Cancer magister* (*C. magister*) and *Ceriodaphnia dubia* (*C. dubia*)), one rotifera (*Brachionus calyciflorus* (*B. calyciflorus*)), one annelid (*Tubifex tubifex* (*T. tubifex*)), two insects (*Chironomus riparius* (*C. riparius*) and *Cloeon dipterum* (*C. dipterum*)), one fish (*Cyprinodon variegatus* (*C. variegatus*)), and one plant (*Selenastrum capricornutum* (*S. capricornutum*)). Table 2a, Table 2b, Table 2c, Table 2d, Table 2e, Table 2f lists the toxicity data (EC_50_ or LC_50_ (μg⋅L^−1^)) of six pesticides for multiple species. The pesticides are acetochlor (ACE), chlorpyrifos (CHL), dimethoate (DIM), glyphosate (GLY), malathion (MAL), and paraquat (PAR).

## 2 Experimental design, materials, and methods

### 2.1 Dataset

The acute toxicity data of multiple chemicals for *X. laevis*, *B. b. japonicus*, *C. magister*, *B. calyciflorus*, *C. dubia*, *T. tubifex*, *C. riparius*, *C. dipterum*, *C. variegatus*, and *S. capricornutum* are 35, 40, 27, 30, 33, 20, 26, 30, 41, and 39, respectively (see Table 1a, Table 1b, Table 1c, Table 1d, Table 1e, Table 1f, Table 1g, Table 1h, Table 1i, Table 1j). The toxicity data of the six pesticides (ACE, CHL, DIM, GLY, MAL, and PAR) for various species are 9, 93, 49, 35, 26, and 28, respectively (see Table 2a, Table 2b, Table 2c, Table 2d, Table 2e, Table 2f). The values of EC_50_/LC_50_ (μg⋅L^−1^) provided in Table 1a, Table 1b, Table 1c, Table 1d, Table 1e, Table 1f, Table 1g, Table 1h, Table 1i, Table 1j were collected from the PAN pesticide database [2] or the United States Environmental Protection Agency ecotoxicology (ECOTOX) database [3]. The data provided in Table 2a, Table 2b, Table 2c, Table 2d, Table 2e, Table 2f include 1) EC_50_/LC_50_ (μg⋅L^−1^) predicted by the developed QSAR models and 2) those from the PAN pesticide database [2] or the ECOTOX database [3].

### 2.2 QSAR prediction

Various molecular descriptors including two-dimensional (2D) topological indices, constitutional indices, ring descriptors, walk and path counts, CATS 2D, and 2D atom-pair descriptors were computed by DRAGON (version 7) [4,5]. Further, variable selection and optimization were performed using the VIPLS [6] and VSMP programs [7]. For more details, refer to the original article, “*Conlecs: A novel procedure for deriving the concentration limits of chemicals outside the criteria of human drinking water using existing criteria and species sensitivity distribution based on quantitative structure-activity relationship prediction*” [1]. Ten robust QSAR models were developed by the above method and the acute toxicities of six pesticides for some species were predicted by the ten developed QSAR models. The values of the optimal descriptors in the QSAR models are listed in Table 1a, Table 1b, Table 1c, Table 1d, Table 1e, Table 1f, Table 1g, Table 1h, Table 1i, Table 1j, and the values of the acute toxicities predicted by the models are provided in Table 2a, Table 2b, Table 2c, Table 2d, Table 2e, Table 2f.
